# Onecut1 and Onecut2 Play Critical Roles in the Development of the Mouse Retina

**DOI:** 10.1371/journal.pone.0110194

**Published:** 2014-10-14

**Authors:** Jillian J. Goetz, Gregory M. Martin, Rebecca Chowdhury, Jeffrey M. Trimarchi

**Affiliations:** Department of Genetics, Development and Cell Biology, Iowa State University, Ames, Iowa, United States of America; Universidade Federal do ABC, Brazil

## Abstract

The entire repertoire of intrinsic factors that control the cell fate determination process of specific retinal neurons has yet to be fully identified. Single cell transcriptome profiling experiments of retinal progenitor cells revealed considerable gene expression heterogeneity between individual cells, especially among different classes of transcription factors. In this study, we show that two of those factors, Onecut1 and Onecut2, are expressed during mouse retinal development. Using mice that are deficient for each of these transcription factors, we further demonstrate a significant loss (∼70–80%) of horizontal cells in the absence of either of these proteins, while the other retinal cells appear at normal numbers. Microarray profiling experiments performed on knockout retinas revealed defects in horizontal cell genes as early as E14.5. Additional profiling assays showed an upregulation of several stress response genes in the adult Onecut2 knockout, suggesting that the integrity of the retina is compromised in the absence of normal numbers of horizontal cells. Interestingly, melanopsin, the gene coding for the photopigment found in photosensitive ganglion cells, was observed to be upregulated in Onecut1 deficient retinas, pointing to a possible regulatory role for Onecut1. Taken together, our data show that similar to Onecut1, Onecut2 is also necessary for the formation of normal numbers of horizontal cells in the developing retina.

## Introduction

Cell fate determination is an intricate process that is controlled by combinations of genes, which have not yet been fully identified. A better understanding of how cellular diversity arises in vertebrates can help us comprehend precisely how cells become specialized to perform specific functions within a complex tissue. Diversity is an especially critical component of the central nervous system’s ability to perform complex tasks such as sensory processing. Developing neural progenitors must integrate extrinsic signals from their environment and neighboring cells, as well as intrinsic cues (usually in the form of transcription factors), in order to make appropriate cell fate determinations. The manner in which the progenitor cell in question interprets these signals varies in different tissues [1], [2]. In a deterministic model of cell fate acquisition, these signals act to facilitate the generation of specific repertoires of daughter cells in a stereotyped fashion [3], [4]. Conversely, a stochastic or probabilistic model of cell-fate determination proposes a more fluid development, wherein the dynamic nature of intrinsic and extrinsic signals lead to changing probabilities of a progenitor cell generating various different cell types [3], [5]. Although evidence for theories of neurogenesis exists in different model systems [5], [6], recent studies in zebrafish suggest that stochastic probabilities play a role in the cell fate determination of the developing retina [3].

The developing retina is an excellent model to study neural cell fate determination due to its relatively simple organization and ease of accessibility. During retinogenesis, a combination of intrinsic and extrinsic signals drives a common pool of retinal progenitor cells to generate a functioning tissue with the correct proportions of six different neurons and one glial cell type [4], [7], [8]. The mature retina is organized into three cell layers: an outer nuclear layer (ONL) consisting of the two types of photoreceptors, rods and cones; an inner nuclear layer (INL) containing horizontal, bipolar, and amacrine interneurons; and lastly, a retinal ganglion cell layer (GCL) composed of displaced amacrine cells and ganglion cells, whose long axons comprise the optic nerve and communicate visual signals to the cortex [9]. During development each retinal cell type is generated at overlapping yet distinctive timepoints from a common pool of progenitor cells [7]. This timeline of generation is stereotypical among vertebrates, with ganglion cells generated first, followed closely by early-born amacrine cells, horizontal cells, and cone photoreceptors [10]–[14]. These early-generated cells are followed by the production of the later-born bipolar cells and the sole retinal glia type, the Muller glia, while the large population of rod photoreceptors is generated throughout retinal development [11], [13]–[15]. Identifying the factors that drive retinal progenitor cells to one cell fate versus another can be challenging, especially when attempting to focus on those that drive the generation of rare yet functionally critical neurons, such as ganglion cells or horizontal cells. Additionally, even retinal progenitor cells that will eventually produce the same type of neuron may be at various stages of development at any given point during retinogenesis. Whole-tissue approaches aimed at uncovering the transcriptomes of progenitor cells during cell fate determination can drown out the signals of rare cells or dynamic changes within certain progenitor cells at various stages of development. Therefore, a single-cell approach was previously utilized to profile the transcriptomic signatures of individual progenitor cells from multiple stages of mouse development [16]. These single cell transcriptomes revealed considerable gene expression heterogeneity among retinal progenitor cells, especially when transcription factors were specifically examined.

One intrinsic factor that is important for retinal development, Math5, is a basic helix-loop-helix (bHLH) transcription factor that is expressed in subsets of retinal progenitor cells in the embryonic retina [17], [18]. Lineage tracing studies have shown Math5 expression in progenitor cells that generated early-born retinal cell types, including rod and cone photoreceptors, amacrine cells, horizontal cells, and ganglion cells [19], [20]. Loss of Math5 leads to an 80–90% decrease in retinal ganglion cells in mice and a complete loss of ganglion cells in the zebrafish [21]–[23]. Concomitantly, the absence of this transcription factor led to an increase in the number of cone photoreceptors and subsets of amacrine cells [21], [23], indicating that it plays a critical role in the normal development of multiple cell types. Given the expression of Math5 and its importance in retinal development, the transcriptomes of single cells expressing Math5 were examined to identify genes involved in the acquisition of early retinal cell fates. These single cell profiles were derived from both progenitor cells isolated at random [16] and targeted cells isolated from Math5-LacZ mice (Trimarchi et al., in preparation).

Transcription factors present in subsets of Math5-expressing (Math5^+^) cells were of particular interest, as they were hypothesized to play a role in the activation of downstream transcriptomic programs that lead to the determination of particular retinal cell fates. Two such factors, Onecut1 and Onecut2, were identified through their expression in subsets of Math5^+^ cells. Additionally, these factors have recently been shown in a separate study to be expressed during retinal development [24]. The Onecut transcription factor family contains three members named for their characteristic structure that includes a single cut domain and a homeodomain [25], [26]. Previously, the Onecut transcription factors have been demonstrated to play critical roles in the development of the liver, bile duct, pancreas and the immune system [25], [27], [28]. In this study, two members of the Onecut family, Onecut1 (OC1) and Onecut2 (OC2), were found to be correlated with Math5 expression in the transcriptomes of individual retinal progenitors. The role of Onecut1 (OC1) in retinal cell fate has recently been investigated [29], but the functionality of its family member Onecut2 (OC2) in the developing retina has not been explored. In this study we examined the retinal phenotypes resulting from the loss of either OC1 or OC2 in mice. In mice deficient for either factor, we observed a significant decrease in horizontal cells, while the other retinal cell populations remained grossly the same. Through the use of microarray profiling, we further characterized both OC1 and OC2 deficient mice. Our results suggest that these transcription factors have significant redundancy in their regulation of horizontal cell genes and reveal possible differences in the regulation of genes in other retinal cell lineages.

## Materials and Methods

### Ethics statement

All procedures for the care and housing of mice conform to the U.S. Public Health Service Policy on the Humane Care and Use of Laboratory Animals and were approved by the Institutional Animal Care and Use Committee at Iowa State University.

### Tissue preparation

Whole eyes were removed and fixed in 4% paraformaldehyde/phosphate buffered saline (PBS) at 4°C for 1 hour. Following fixation, eyes were rinsed 3 times in PBS at 4°C for 10 minutes. The sclera was dissected away from the intact lens and retina. Retinas (with lens intact) were post-fixed in 4% paraformaldehyde/PBS at 4°C for 15 minutes and then rinsed 3 times with PBS. The lens was removed and the retina taken for further processing depending upon the particular assay.

### Whole-mount immunohistochemistry

Dissected retinas were equilibrated at 4°C in sucrose solutions of increasing concentrations in a step-wise manner, 10%, 20%, 30% sucrose (w/v) in PBS, for at least 30 minutes or until the retina had settled to the bottom of the tube. While the retinas were in 30% sucrose/PBS, they were snap-frozen on dry ice and subjected to three freeze/thaw cycles. Retinas were stored at −80°C in the 30% sucrose/PBS solution or used for immunostaining immediately. Following freeze/thaw, retinas were rinsed 3 times in PBS for 30 minutes and blocked for 2 hours at room temperature (RT) in blocking solution [3% goat serum/1% bovine serum albumin (BSA)/0.1% Triton-X100/0.02% sodium dodecyl sulfate (SDS) in PBS]. Retinas were then incubated in primary antibody in blocking solution overnight at 4°C on a rocking platform. The next day, retinas were rinsed 3 times in PBS for 30 minutes and placed in secondary antibody in blocking solution overnight at 4°C on a rocking platform. Retinas were rinsed 3 times in PBS for 30 minutes and then flattened between two coverslips for confocal imaging on a Leica SP5 X MP confocal microscope.

### Tissue sectioning

Dissected retinas were equilibrated in 30% sucrose/PBS at 4°C until they sank to the bottom of the tube. They were then frozen in an equilibrated solution of 50% (30% sucrose/PBS): 50% optimal cutting temperature compound (OCT, Tissue-Tek; Sakura Finetek, Torrance, CA) and stored at −80°C. Cryosections were cut at 20 µm, collected on Superfrost Plus slides (Fisher Scientific), and air-dried for 30 minutes at RT before storage at −80°C.

### Section antibody staining

Sections were incubated in blocking solution (1% BSA/0.1 Triton-X100 in PBS) for 1 hour at RT and then in primary antibody in blocking solution overnight at 4°C. Slides were rinsed in blocking solution 3 times and incubated with secondary antibody in blocking solution for 2 hours at RT. Slides were rinsed with PBS, mounted in Fluoromount-G and visualized on a Leica SP5 X MP confocal microscope.

Primary antibodies used were anti-Calbindin28K (1∶2000) (Swant, Switzerland), anti-Chx10 (1∶1000) [30], anti-Glutamine Synthetase (1∶10,000) (Sigma), anti-Rhodopsin (1∶100) [31], anti-GFAP (1∶100) (Abcam), and anti-HNF-6/OC1 (1∶200) (Santa Cruz Biotechnology). The anti-Pax6 (1∶50) and anti-Islet1 (1∶50) antibodies were obtained from the Developmental Studies Hybridoma Bank (DHSB), developed under the auspices of the NICHD and maintained by the University of Iowa, Department of Biology, Iowa City.

### Quantitative immunohistochemical analysis

For analysis, identically-sized fields of immunostained retinal cryosections were counted by hand in Adobe Photoshop, blinded to genotype. After counts were made for WT and KO fields (n = 3 biological replicates for both - except in the case of Calb28k, in which 4 fields of both amacrine cell staining and horizontal cell staining were counted), t-tests were performed to compare counts of the WT and KO fields. Error bars indicate standard error.

### Section in situ hybridization

#### Riboprobe synthesis

Probe template sequences were amplified from mouse cDNA, cloned into the pGEM-T vector (Promega) and sequenced. Antisense riboprobes were transcribed using either T7 or Sp6 RNA polymerase in the presence of digoxigenin (DIG) or fluorescein-labeled nucleotides for 1–2 hours at 37°C. Riboprobes were treated with DNase I for 15 minutes at 37°C and precipitated overnight with LiCl and 100% ethanol.

#### *In Situ* Hybridizations (ISH)

*In situ* hybridizations on retinal cryosections and dissociated cells were performed exactly as described in Trimarchi et al. 2007. For the dissociated ISH, one probe was synthesized with a digoxigenin (DIG)-label and the other probe was labeled with fluorescein. DIG-labeled probes were detected using an anti-DIG-POD antibody (Roche, 1∶1000) and a Cy3 tyramide solution (PerkinElmer), while fluorescein-labeled probes were detected using an anti-fluorescein-POD antibody (Roche, 1∶1000) and an Alexa-488 tyramide (Life Technologies). Tyramide amplification (Life Technologies) was performed for 10 minutes for the DIG-labeled probe, followed by inactivation in 0.3% hydrogen peroxide and tyramide amplification for the fluorescein-labeled probe. The slides were fixed in 4% paraformaldehyde and mounted. Six independent fields were photographed and quantified for each.

### Microarray experiments

#### Total RNA Isolation

RNA was extracted from retinal tissue using TRI-Reagent (Sigma) according to the manufacturer’s instructions. Briefly, tissue was homogenized in 1 ml of TRI-Reagent, 0.1 ml of 1-bromo-3-chloropropane was added to sample, followed by vigorous shaking for 15 seconds. Samples were allowed to sit at RT for 10 minutes and centrifuged at 13,300 rpm for 15 minutes at 4°C, and the resulting aqueous phase was isolated. RNA was precipitated by adding 0.5 ml of isopropanol per 1 ml of TRI-Reagent and allowing the sample to incubate at RT for 10 minutes followed by centrifugation at 13,300 rpm for 10 minutes at 4°C.

#### RNA Amplification

Total RNA from retinal tissue was amplified, biotinylated, and fragmented using the MessageAmp III RNA Amplification Kit (Ambion), following the manufacturer’s instructions. All steps were carried out in a thermal cycler. 10 µg of amplified RNA was fragmented for all of the directly compared samples. Amplified RNA was hybridized to Affymetrix GeneChip Mouse Genome 430 2.0 arrays at the Iowa State University GeneChip Facility. Microarray analysis was performed using the Affy R package developed by Bioconductor. After background adjustment and normalization using Mas5, the data were log(2) transformed. To be considered for differential expression analysis, the log(2) transformed mean of either n = 3 WT or n = 3 KO expression values must have exceeded 7 to indicate overall expression in either genotype. A two-tailed t-test which resulted in p-values of less than 0.05 indicated significant differential expression.

### qPCR

RNA was isolated from individual mouse retinas at various ages using TRI-reagent (Sigma). cDNA was generated from 400 ng of retinal RNA using random primers and SuperScript III (Life Technologies) according to the manufacturer’s instructions. qPCR was performed using SYBRGreen MasterMix (Thermo) and a BioRad CFX96 Real Time System with BioRad C1000 Thermal Cycler using the following program: 15′ at 95°C and 40 cycles of 15 sec at 95°C, 30 sec at 56°C, and 30 sec at 72°C. Each sample was normalized to β-Actin primers. The analysis of the qPCR data was performed exactly as in Livak and Schmittgen (Livak and Schmittgen, 2001). Briefly, the difference in average ΔΔC(t) values and the difference plus and minus the standard error of the difference were computed on the C(t) scale. The base-2 antilogs of these three values were computed to obtain values with error bars on the fold change scale. Results were plotted on a logarithmic scale.

### Mouse genotyping

OC1 and OC2 deficient mice were genotyped with primer pairs for the WT and null alleles. OC1-WT: 5′-CAGCACCTCACGCCCACCTC-3′, 5′-CAGCCACTTCCACATCCTCCG-3′; OC1-KO: 5′-CTGTGCTCGACGTTGTCACTG-3′, 5′-GATCCCCTCAGAAGAACTCGT-3′. OC2-WT: 5′-GCCACGCCGCTGGGCAAC-3′, 5′-CAGCTGCCCGGACGTGGC-3′; OC2-KO: 5′-GACCGAGTACAAGCCCACG-3′; 5′-GTCCGCGACCCACACCTT-3′. Reactions containing all four primer pairs for each mouse strain were amplified by 35 cycles of PCR, with the same program for both strains (94°C for 30 seconds, 60°C for 30 seconds, 72°C for 30 seconds, with a 3 minute initial denaturation at 94°C). OC1 PCR reactions included WT primers at 0.2 µM each and null allele primers at 1.6 µM each. OC2 PCR reactions included all WT and null allele primers at 0.8 µM, as well as 1X MasterAmp (Epicentre). OC1 genomic DNA was isolated by adding 200 ul 50 mM NaOH to tissue samples (ear clips for live mice, confirmed by tail clips upon harvesting) and incubating at 95°C for 30 minutes, followed by the addition of 50 ul 1 M Tris pH 8 and centrifugation for six minutes at 13,300 rpm at 4°C. OC2 genomic DNA preparation required phenol-chloroform (1∶1) extraction followed by ethanol precipitation.

## Results

**Figure 1:**
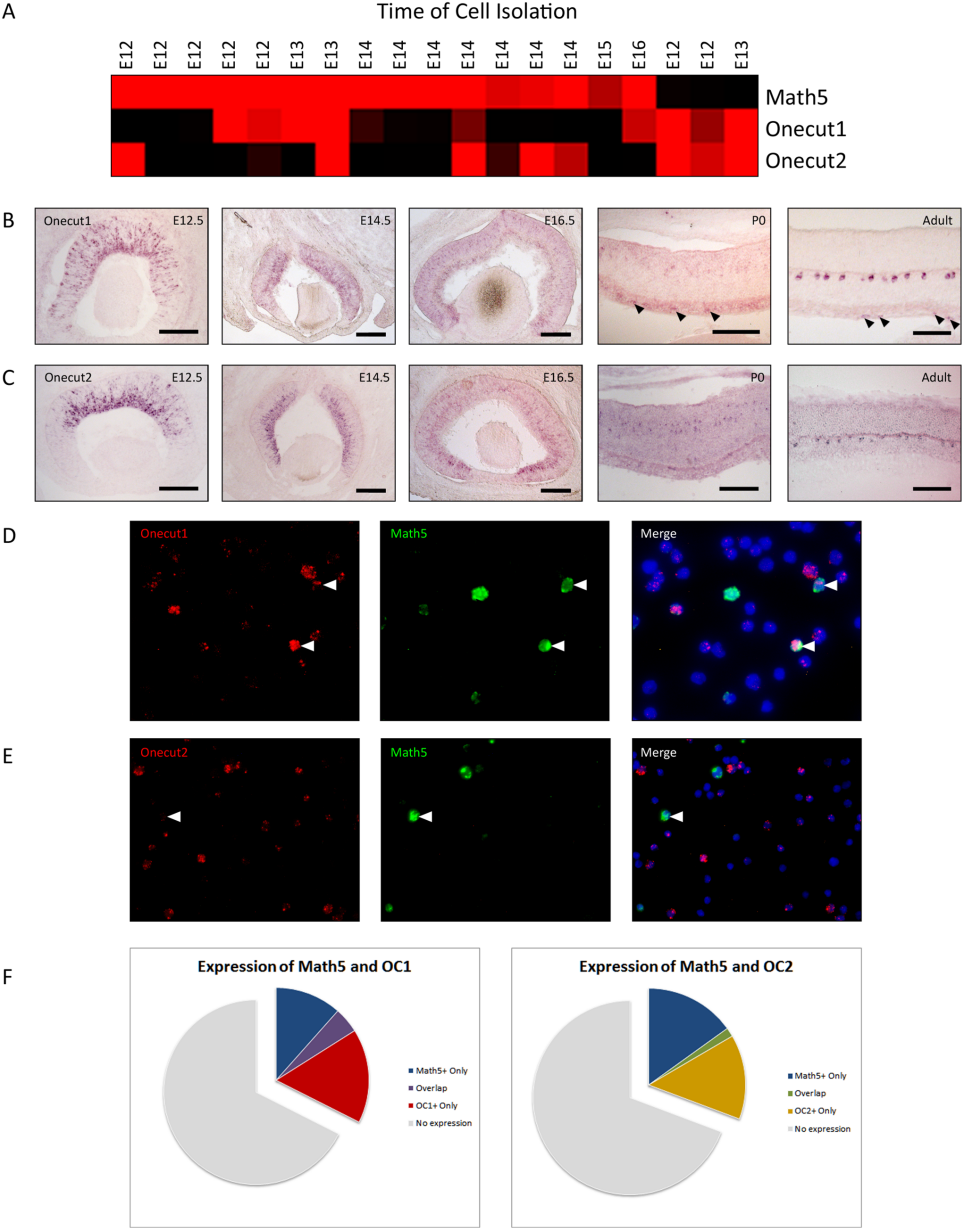
Expression of Onecut1 and Onecut2 in the developing murine retina. The expression of Onecut transcription factors was analyzed throughout retinal development (A). A heatmap indicating the genes (rows) expressed in isolated single retinal progenitor cells (columns) at various stages of development, from embryonic day (E)12.5 to E16.5. Increased expression of a gene in a given cell is indicated in shades of red, while the absence of expression is indicated with a black square. Expression patterns of Onecut1 (B) and Onecut2 (C) mRNAs were determined via in situ hybridization at various stages of retinal development and in the adult retina. Arrowheads indicate expression in the ganglion cell layer. Adult scale bars represent 100 µm; all others 200 µm. (D,E) Dissociated cell in situ hybridization was performed at E14.5 using a probe targeting either Onecut1 or Onecut2 and Math5. Arrowheads indicate overlapping Math5 and Onecut family member expression. (F) Quantification of dissociated retinal cells expressing Math5, Onecut1, and Onecut2.

The Math5 transcription factor is expressed during the early stages of mouse retina development in the lineages of many retinal cells, including photoreceptors, amacrine cells, horizontal cells, and ganglion cells [20]. To identify genes involved in the cell fate determination process of early-born retinal neurons, we isolated single Math5^+^ progenitor cells and identified genes that were strongly correlated with Math5 expression in these cells [16]. Microarray data revealed that members of the Onecut family of transcription factors (Onecut1 and Onecut2) were present in subsets of Math5^+^ single cells (Figure 1A). To confirm the findings of the single-cell microarrays, as well as to better understand the expression of these factors in the retina, *in* *situ* hybridization (ISH) was performed for Onecut1 (OC1) (Figure 1B) and Onecut2 (OC2) (Figure 1C) at various timepoints during retinal development. Both transcription factors were observed in subsets of retinal cells, in an area consistent with expression in retinal progenitor cells and newly generated retinal neurons (Figure 1B, C). To quantify the number of cells that co-expressed Math5 and the Onecut factors, dissociated fluorescent ISH was utilized (Figure 1D, E). At E14.5, a significant percentage (27.2%) of Math5^+^ cells also expressed OC1, while 10.6% of Math5^+^ cells also showed expression of OC2 (Figure 1F). As the retina matured, the expression of both OC1 and OC2 became limited to the apical side of the inner nuclear layer, where the horizontal interneurons are located (Figure 1B, C). In addition, OC1 expression was observed in a subset of cells in the GCL (Figure 1B). Interestingly, only OC1 expression was observed in the GCL and not OC2, whereas both proteins were previously reported to be in the GCL by antibody staining (Wu et al., 2012). This may reflect a difference in mRNA and protein expression. Otherwise, the timing and location of expression of Onecut1 and Onecut2 is consistent with a recent publication [24] and suggests a possible role for the Onecut transcription factors in regulating retinal development. With these data in mind, we obtained OC1 and OC2-deficient mice (Clotman, et al., 2005) and determined how loss of these transcription factors affected retinal cell populations.

### Retinal phenotypes in the OC1-deficient mouse

**Figure 2:**
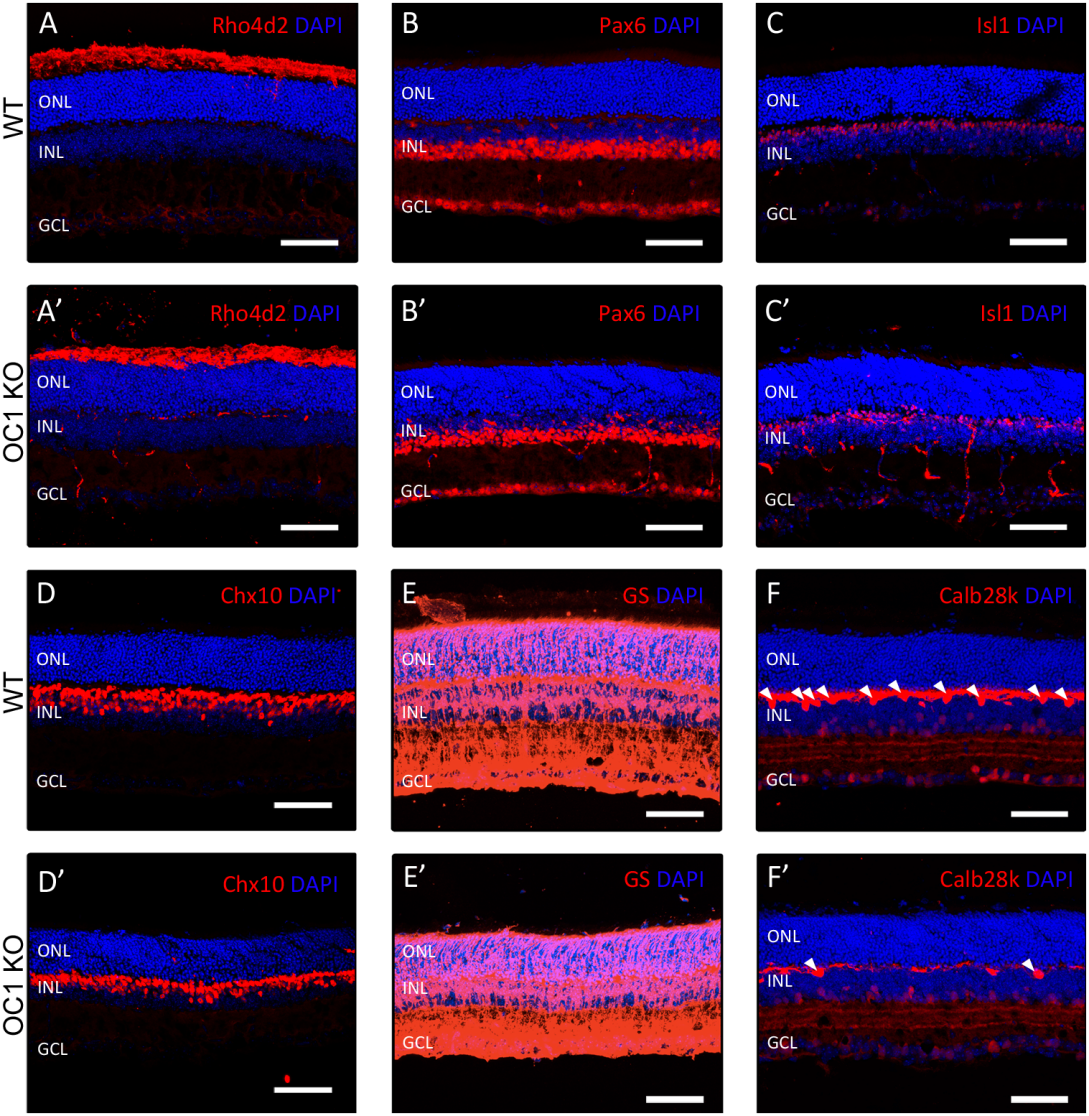
Immunohistochemistry of Adult OC1 Knockout Retinas. Changes in mature retinal cell populations resulting from the loss of OC1 were examined using immunohistochemistry. Most retinal populations were unchanged between wildtype and OC1-KO littermates, including rod photoreceptors (A,A’), amacrine cells (B,B’,C,C’), bipolar cells (D,D'), and Muller glia (E,E’). However, anti-Calb28k-staining shows a decrease in horizontal cells in OC1-KO retinas as compared to a wildtype littermate (F,F'). Arrowheads indicate horizontal cell nuclei. Scale bars represent 100 µm.

Previous studies have demonstrated that OC1 is a critical transcription factor in liver development (Clotman, et al., 2002). Due to this important function, most OC1-KO mice die either at birth or in the immediate postnatal period. Despite the high mortality rate resulting from complete loss of OC1, we were able to obtain a few mature, postnatal day (P)21 OC1-KO mice. Since the effects of OC1 deficiency on retinal development had not previously been studied in this particular mouse line, we examined whether mature retinal cell populations were present in the absence of OC1. To specifically determine any deficits in populations of retinal cells, P21 OC1-KO retinas were surveyed using immunohistochemistry with antibodies that mark each subset of retinal cells (Figure 2). Compared to wildtype littermate control animals (Figure 2A–E), populations of rod photoreceptors (Figure 2A’), amacrine cells (Figure 2B’), bipolar cells (Figure 2C’,D’), and Muller glia (Figure 2E’) were unchanged in adult OC1-KO mice. OC1 has previously been shown to play an important role in the development of horizontal cells using a conditional knockout mouse model [29]. Consistent with this report, a significantly decreased horizontal cell population was readily apparent in our P21 OC1-deficient mice (Figure 2F, F’).

### Retinal phenotypes in the OC2 deficient mouse

**Figure 3:**
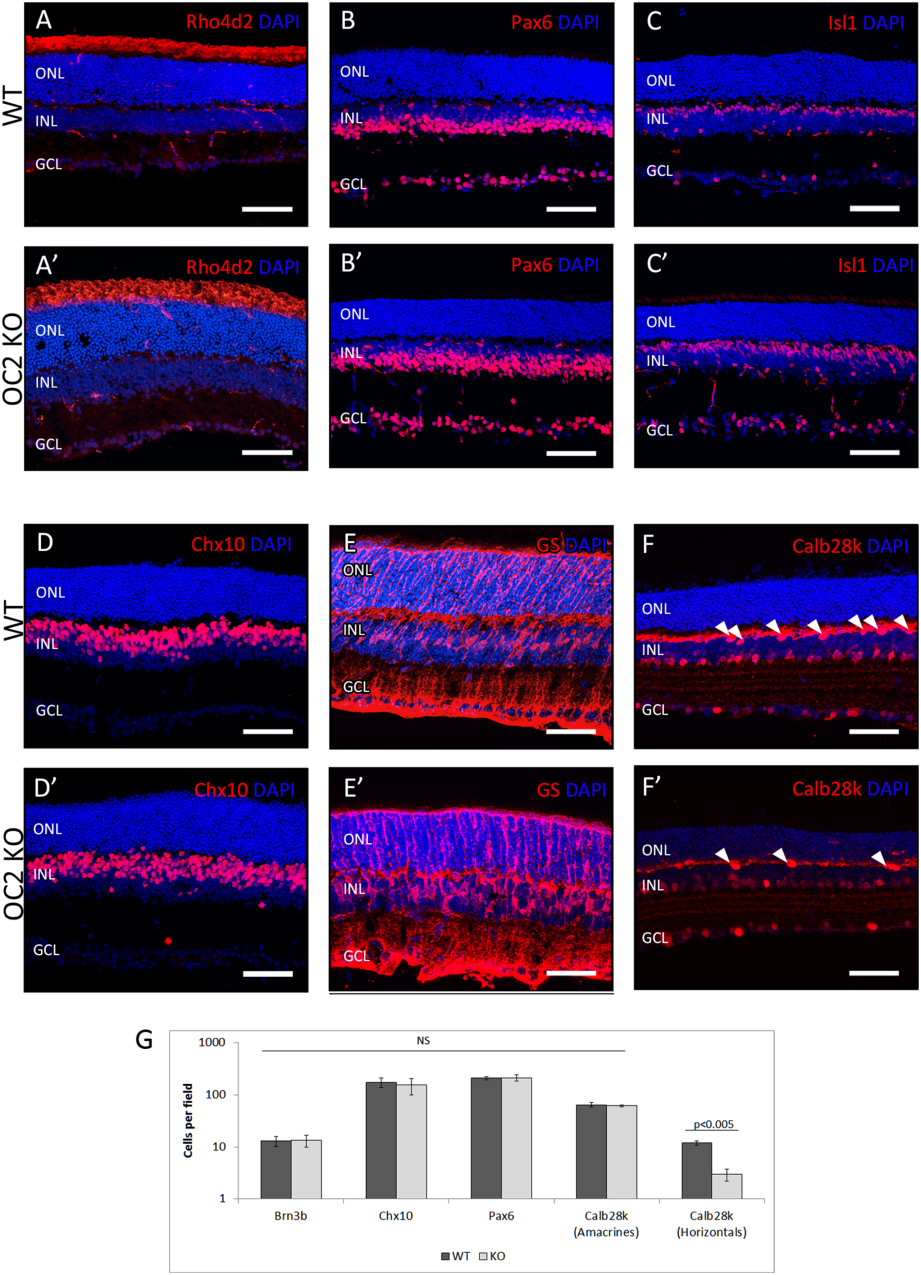
Immunohistochemistry of Adult OC2 Knockout Retinas. Changes in mature retinal cell populations resulting from the loss of OC2 were examined using immunohistochemistry. Rod photoreceptors (A,A'), amacrine cells (B,B',C,C'), bipolar cells (D,D'), and Muller glia (E,E') were unchanged, whereas the horizontal cell population was greatly decreased (F,F'; arrowheads indicate horizontal cell bodies). Scale bars represent 100 µm. (G) The results of the immunohistochemical analyses were quantified for identically-sized fields of cryosectioned retinal tissue. Although staining for Brn3b, Chx10, Pax6, and the Calb28k that marks amacrine cell bodies were unchanged, horizontal cell bodies marked by Calb28k were significantly decreased in OC2-KO retinas (n = 3, p<0.005).

We observed that OC2 is expressed at developmental timepoints and in cell populations reminiscent of its family member, OC1 (Figure 1). Therefore, we next explored the consequences resulting from the loss of OC2 during retinal development. To determine whether the development of retinal cell populations was disrupted by loss of OC2, adult (P21) retinal cell populations of OC2-deficient mice were surveyed by immunohistochemistry using a similar panel of antibodies as in the OC1-deficient mouse retinas (Figure 3). No changes were observed in populations of rod photoreceptors (as marked by αRho4d2, Figure 3A, A’), amacrine interneurons (αPax6, Figure 3B, B’), bipolar cells (αIsl1, Figure 3C, C’; αChx10, Figure 3D, D’), or Muller glia (αGS, Figure 3E, E’). However, horizontal cell populations were significantly decreased in OC2-KO, to a similar degree as observed in the OC1-KO (Figure 3F, F’). To ensure there were not any small, but significant differences in these cell types, cell counts were performed on sections from n = 3 different wildtype and OC2-KO mice. No significant differences were observed for any cell types except the horizontal cells (Figure 3G). To further confirm the results obtained through immunohistochemical analysis, we performed *in* *situ* hybridizations on cryosections from P21 OC2-KO retinas. We did not observe any significant differences between OC2-KO animals and their wildtype littermates in either the rod or cone photoreceptor populations (Figure S1 A–C’), bipolar cells (Figure S1D,D’), GABAergic (GAD1– Figure S1E,E’) and glycinergic (SLC6A9– Figure S1F,F’) amacrine interneurons (Figure S1G,G’,H,H’), ganglion cells (Figure S1I,I’), and Muller glia (Figure S1J,J’). However, ISH did reveal a significant decrease in horizontal cells (Figure S1K,K’,L,L’).

**Figure 4:**
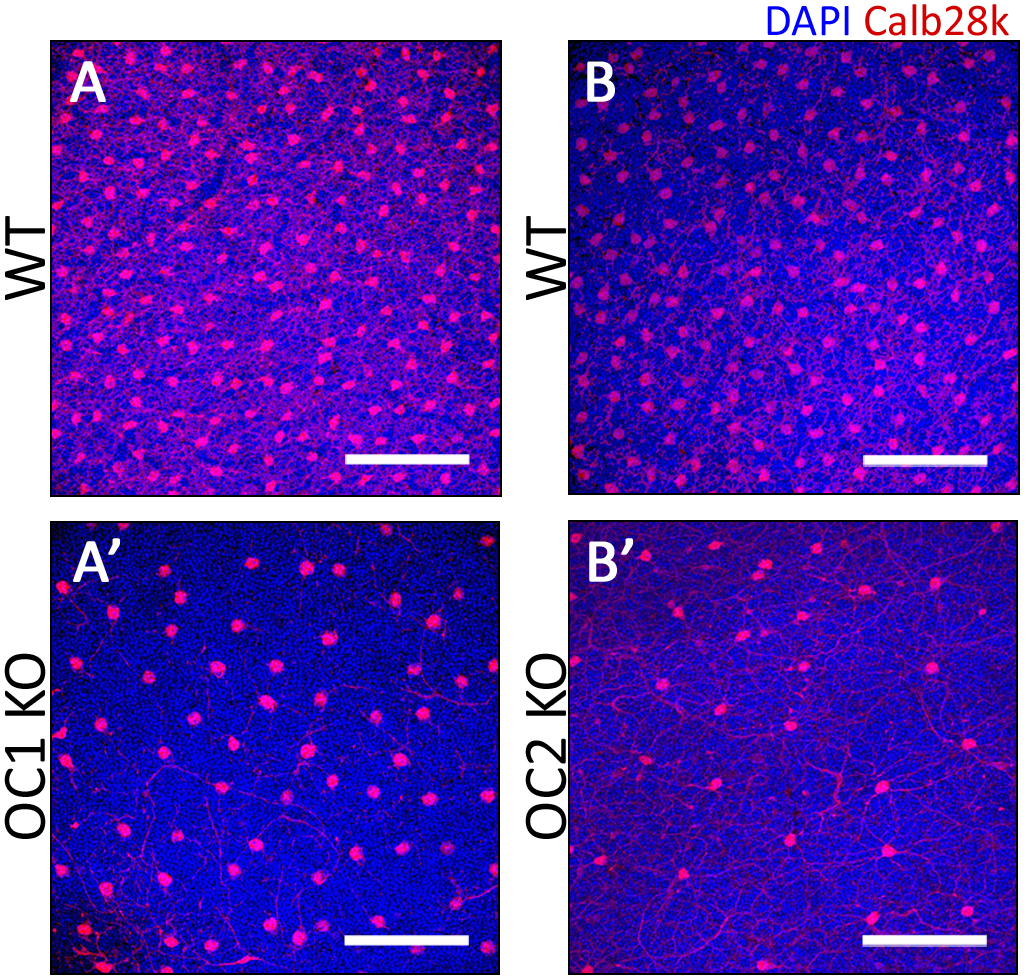
Assessment of horizontal cells by flat mount retina staining. To better understand the distribution of horizontal cell loss in the OC1 and OC2-KO retinas, immunohistochemistry was performed using an anti-Calbindin 28k antibody on age-matched WT (A,B), OC1-KO (A’), and OC2-KO (B’) retinas. Scale bars represent 100 µm.

To gain perspective on the overall patterning of horizontal cell loss, whole, flat-mounted retinas were stained using an antibody against the horizontal cell marker Calbindin-28k (Calb28k) (Figure 4). Whole mount staining showed a significant loss of the horizontal cell population across the entire retinas of both the OC1 (Figure 4A, A’) and OC2-deficient mice (Figure 4B, B’). In P21 OC2-deficient retinas, horizontal cells were decreased to about 20% of the levels seen in wildtype littermates (n = 3, p = 2e^−7^). Given the difficulty of obtaining OC1 mice at maturity we were only able to quantify one animal, but the horizontal cells were decreased by 35%. These observations demonstrate show that loss of either Onecut1 or Onecut2 interferes with either the generation or maintenance of horizontal cells in the murine retina.

In a previous study, Onecut1 deficient retinas were found to exhibit degeneration of the photoreceptor layer (ONL) by 8 months of age [29]. Therefore, we examined retinas of OC2-deficient mice for possible ONL degeneration at two different ages (Figure S2). Staining for Calbindin-28k indicated that the decrease in horizontal cells in OC2-KO mice compared to WT mice was maintained well into maturity (Figure S2). However, antibody staining for rod photoreceptors in the OC2-KO appeared grossly normal at 12 months of age when compared to a wildtype littermate (Figure S2A,A’). Conversely, a 16-month old OC2-deficient mouse displayed a thinner layer of rhodopsin staining compared to a WT age-matched littermate (Figure S2B,B’). These results indicate that although the horizontal cells are similarly decreased in both Onecut1 and Onecut2 deficient mice, the photoreceptor degeneration is much slower and less pronounced in the OC2-KO mouse compared to the OC1-KO.

**Figure 5:**
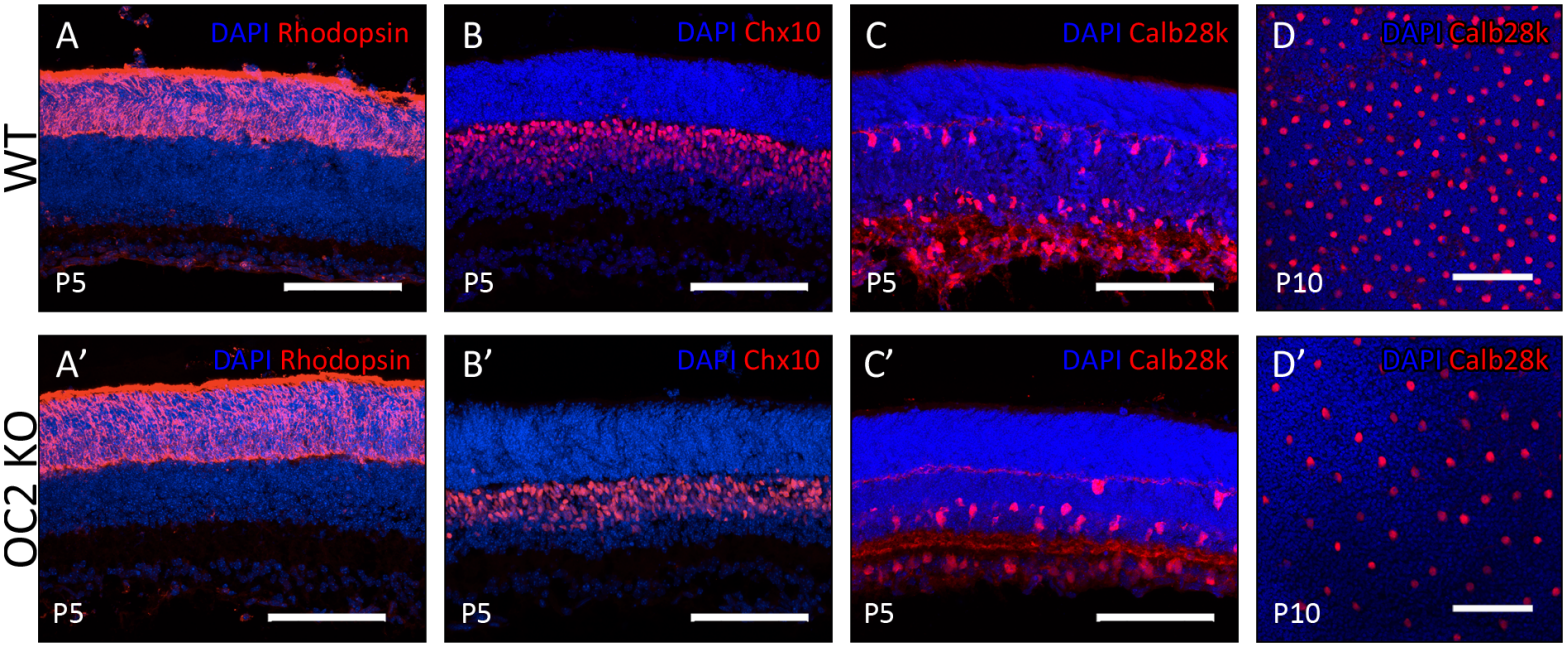
Examination of early postnatal stages in OC2-KO retinas. To determine whether this decrease in horizontal cells was confined to the fully mature retina, or was present earlier in development, immunohistochemistry was performed on the early postnatal retina. Age-matched animals were processed for either retinal sections (P5) or flatmounts (P10) and stained with an anti-Rhodopsin antibody (A,A’), an anti-Chx10 antibody (B,B’), or an anti-Calbindin 28k antibody (C,C’,D,D’). Scale bars represent 100 µm.

We have observed that mature mouse retinas are either unable to generate or maintain wildtype levels of horizontal cell populations in the absence of either OC1 or OC2. To better understand the developmental stage when this phenotype becomes apparent, we investigated OC2 deficient retinas at various stages of maturity, starting with P5–10 (Figure 5). At these stages, nearly all retinal cells, including horizontal cells, are postmitotic [13]. Immunohistochemical analysis showed that at P5, the development of other retinal cells, including the late-developing rod photoreceptors (αRho, Figure 5A, A’) and bipolar cells (αChx10, Figure 5B, B’) appear normal. However, postnatal retinas lacking Onecut2 already show a marked decrease in horizontal cells both at P5 (Figure 5C, C’) and at P10 (Figure 5D, D’). These results suggest that the loss of horizontal cells in OC2-deficient retinas may not be due to a lack of horizontal cell maintenance, at least in the postnatal stages of development.

It is apparent that horizontal cell populations are disrupted before retinal maturation is completed in OC2-KO retinas. However, it is unclear whether the progenitor cells that would normally become horizontal cells undergo a fate change and become other retinal cell types, or if they differentiate normally into horizontal cells only to die in the absence of OC2. Horizontal cells are among the earliest born cell types in the retina, so a cell-fate change might be predicted to affect the population sizes of other early-born neurons. To examine whether a cell fate change was apparent in early retinal differentiation, ISH was performed at E16.5 (Figure S3). A high percentage of early-born retinal cells including cone photoreceptors, some amacrine interneurons, and ganglion cells were generated by this late embryonic stage. There were no significant differences in progenitor cells at E18.5 as visualized by Chx10 (Figure S3A,A’), photoreceptor precursor cells (Prdm1, Figure S3B,B’; Otx2, Figure S3C,C’; Gnb3, Figure S3D,D’), amacrine cell precursors (Tcfap2b, Figure S3E,E’), and developing ganglion cells (Sncg, Figure S3F,F’) in the absence of OC2.

Although changes in early-born retinal populations are not apparent at E16.5 in OC2-KO mice, we were also interested in visualizing the cell types generated in the early stages of retinogenesis in OC1-KO mice. We performed *in* *situ* hybridization at E16.5, a timepoint when horizontal cells, ganglion cells, cone photoreceptors, and amacrine cells are being actively produced (Figure S4). As in the OC2-KOs, no changes were identified during mid-embryonic stages in the developing photoreceptors as visualized using probes to Prdm1, Gnat2, and Gnb3, (Figure S4A,A’–C,C’). Additionally, no significant changes were noted in amacrine cells (Tcfap2b, Figure S4D,D’) or ganglion cells (Sncg, Figure S4E,E’). Horizontal cells are an extremely small population of neurons that are generated over multiple days during murine retinogenesis [15] and are, therefore, difficult to track over the span of their generation. We have not observed any changes in other retina cell populations or indications of increased retinal cell death. However, the effect of the loss of cells or fate-switch in such a small population may not generate statistically significant changes in cell death or cell-fate change at any single point over the developmental timeline.

### Global gene expression changes in Onecut2-deficient mouse retina

To assess global changes in gene expression that were not immediately apparent from our visual inspection of retinal cell populations, we performed microarray analysis on adult OC2 retinas (data available on NCBI GEO: reference number GSE57918). Retinas were dissected from OC2-KO animals and their wildtype littermates and RNA was isolated, then reverse-transcribed to cDNA and hybridized to Affymetrix microarrays. We first examined genes that were downregulated in adult OC2-KO retinas (Table S1). Our prediction was that genes with lower expression would point to any additional cellular deficiencies in the OC2-KO mice. Consistent with our previous observations regarding a significant deficit in horizontal cells in these mice, the genes Calb1 and Septin4 [32] were significantly downregulated (Table S1). Surprisingly, the signals for two bipolar cell genes, Pcp2 and Car8 [33], were also significantly downregulated, pointing to a possible subtle bipolar cell phenotype in the OC2-KO mice.

**Figure 6:**
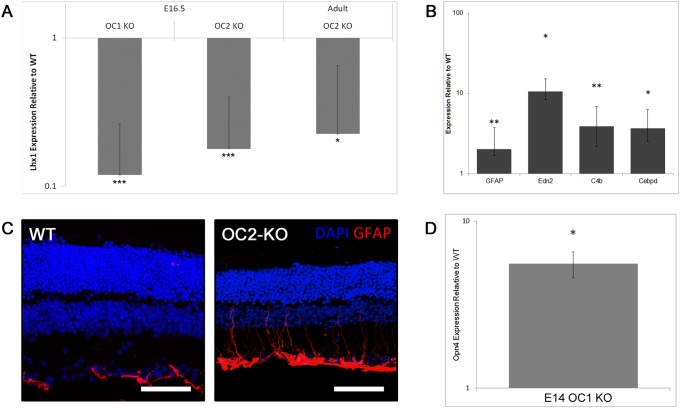
Unbiased search for changes in the OC2-deficient retina. (A) Quantitative PCR (qPCR) analyses determining the relative amounts of Lhx1 in OC1-KO, and OC2-KO retinas at E16.5 or adult compared to WT littermate retinas. (B) qPCR determining the relative amounts of various genes identified in models of retinal damage or disease expressed in adult OC2 retinas. Each marker known to be involved in retinal stress is significantly upregulated in OC2 compared to their WT littermates, including GFAP, Edn2, C4b, and Cebpd. (C) WT and OC2-KO retinas were stained with an anti-GFAP antibody. Scale bars represent 100 µm. (D) qPCR determining the relative amounts of Opn4 in E14.5 OC1-KO retinas compared to WT littermate retinas. * indicates p<0.05, ** indicates p<0.01, *** indicates p<0.005. Scale bars represent 100 µm. All quantitative results are plotted on logarithmic scale.

The LIM1 class transcription factor Lhx1 is specifically expressed in differentiating and mature horizontal cells [34]. We examined our microarray data and found that while the expression of Lhx1 was decreased, it did not reach significance (p = 0.159). To ascertain whether Lhx1 was in fact downregulated in OC2-KO retinas, we performed qPCR and found that Lhx1 expression was significantly decreased in the OC2-KO adult retina (Figure 6A). To further pinpoint when this downregulation occurs, we performed qPCR for Lhx1 on WT and OC2-KO retinas at E16.5. At this timepoint, we also observed that Lhx1 expression was significantly lower in the OC2-KO than in the WT (Figure 6A), suggesting that horizontal cells are decreased in these mice from very early on in development. Interestingly, our data suggests that this regulation of Lhx1 by the Onecut factors may be dosage dependent. Removal of just a single copy of either OC1 or OC2 (data not shown) reduced the amount of Lhx1 present. This result was consistent, but not high enough to reach statistical significance. Together, these microarray and qPCR results point to an early defect in horizontal cells and a possible bipolar cell phenotype in the OC2 deficient mice.

Next, we examined genes that were upregulated in the absence of OC2 (Table S2). Curiously, OC2 was one of the most highly upregulated genes in OC2-KO retinas as compared to their WT littermates. To understand this conundrum, we investigated the location of the sequences used on the microarray and ISH-probes and found that they were located at the 3′ end of the Onecut2 gene. To see if this upregulation was consistent throughout the entire sequence, we targeted the 5′ coding sequence of the Onecut2 gene by qPCR and found it to be virtually undetectable (data not shown). Additionally, OC2 protein as visualized through immunohistochemistry was not observed in the OC2-KO retinas (data not shown). These observations suggest that the remaining leftover OC2 sequence in the OC2-KO mouse is upregulated at the RNA level, but that no functional protein is produced.

Another highly upregulated gene observed in OC2-KO retinas was glial fibrillary acidic protein (GFAP), a marker of activated gliosis [35]. To confirm the upregulation of GFAP, qPCR was performed on OC2-deficient retinas. A twofold upregulation of GFAP was observed (n = 7 WT, n = 9 KO animals; p = 0.002; Figure 6B). Antibody staining for GFAP revealed that the processes of Muller glia have upregulated the protein in OC2-KO retinas when compared with the WT littermates (Figure 6C). To get a greater sense of other changes present in the KO retinas, especially those related to a possible stress response, we explored changes in other genes identified to be upregulated in models of retinal disease. Indeed, further investigation of markers of retinal stress indicated that adult OC2 retinas overexpress numerous genes implicated in stress or injury-related contexts ([36]–[39], such as Edn2, C4b, and Cebpd, in both microarray and follow-up qPCR (Figure 6B). In addition, CD44, a Muller glia expressed gene that is increased upon retinal degeneration [40], is upregulated in OC2-KO retina microarrays (Table S2). Despite the normal rhodopsin staining observed in OC2-KO retinas, the upregulation of these stress response and gliosis-associated genes suggest that retina health is compromised in the absence of OC2 and/or horizontal cells.

To ascertain any developmental phenotypes in the OC1-KO retinas, microarrays were performed on retinas isolated from E14.5 mice (data available on NCBI GEO: reference number GSE57917). Among the genes that were consistently upregulated, two of them stood out. Gephyrin and Sox4 were significantly upregulated in the E14.5 OC1-KO retinas (Table S3). Given the expression of these genes in ganglion and amacrine cells [41], [42], their upregulation may point to a possible subtle phenotype in these cells. One interesting gene that was observed to have a higher, but not quite statistically significant, expression in the E14.5 OC1-KO versus WT was melanopsin (Opn4). Opn4 encodes a photopigment protein that is expressed in a small subset of intrinsically photosensitive retinal ganglion cells [43]. While much research has been done on the function of these cells, very little is known about how these cells are generated and how the Opn4 gene is regulated. Since the differential expression of Opn4 only approached significance (p = 0.08), we examined the expression of Opn4 by qPCR in WT and OC1-KO retinas at E14.5. By this assay we observed a statistically significant upregulation of Opn4 (Figure 6D). Surprisingly, when we examined OC1-KO retinas at slightly later timepoints (E16.5 and E17.5) we no longer observed this upregulation of Opn4 (data not shown); this suggests that other factors may be able to compensate for the loss of OC1. These data identify the Onecut1 transcription factor as a possible repressor of Opn4 expression.

We also examined the genes that were downregulated in E14.5 OC1-KO retinas when compared to WT littermates (Table S4). While most of the genes that were downregulated have unknown retinal functions, two genes, Irx4 and Scratch2, stood out. These two genes have been associated with developing retinal ganglion cells [44], [45], suggesting that there may be subtle defects in retinal ganglion cell development or differentiation in the OC1-KO retinas that warrant further investigation.

## Discussion

In this study, our goal was to better understand the factors that contribute to neurogenesis in the developing murine retina. By profiling the transcriptomes of individual cells expressing Math5, a transcription factor integral to the early stages of retinal development [21]–[23], we identified two genes, Onecut1 (OC1) and Onecut2 (OC2), which were also expressed in the developing mouse retina. Specifically, we found that both OC1 and OC2 were expressed in patterns similar to Math5 in the embryonic retina and later their expression became more confined to mature horizontal cells. An examination of mature retinas from mice deficient for either OC1 or OC2 revealed that loss of either factor led to a marked decrease in adult horizontal cell populations as seen through immunohistochemistry, *in* *situ* hybridization, microarrays, and qPCR analysis. Microarray analysis of OC2-deficient retinas also revealed an upregulation of stress-related genes, such as GFAP, indicative of retinal stress and reactive gliosis. However, significant degeneration of the ONL was not observed until 16 months of age in the OC2-KO mice. Additionally, our microarray profiling has revealed potential phenotypes in subsets of other retinal cell types, which were not observed either in our bulk population antibody staining or in a previous study of a conditional Onecut1 retinal knockout [29]. Overall, our results indicate that both OC1 and OC2 play an important role in the development of horizontal cell populations within the retina, and that the loss of these transcription factors compromises the overall health of the mature retina.

OC1 and OC2 appear to share overlapping roles in the development of the retina. Loss of either gene alone led to a decrease in horizontal cells, although neither deficiency on its own led to a complete lack of these cells. This suggests some amount of redundancy in the functionality of these Onecut family members during retinal development. Our results are consistent with the fact that OC1 and OC2 share a 92% amino acid similarity in their DNA binding domains [28] and that they have been shown to bind to the same consensus DNA binding site [46]. Although embryonic lethality precluded an in-depth analysis of this redundancy, as we were unable to recover viable double-KO mice, we were able to examine mice lacking three of the four OC1 and OC2 alleles by qPCR. These analyses indicated that the changes in expression in the transcription factor Lhx1 are more severe as additional alleles of the Onecut family genes are removed (data not shown). Due to issues of lethality, we were unable to obtain enough animals to perform a rigorous statistical test on this data; nevertheless, the data do suggest a possible dosage-dependent model for Onecut regulation of Lhx1.

The expression of Onecut1 and Onecut2 has been previously examined during retinal development [24]. As in our single-cell studies, the previous association of OC1 and OC2 with Math5 in early retinal development suggested that these genes play a role in the cell-fate determination of early-developing retinal cells. Our findings agree with previous results that OC1 is critical for the development of horizontal cells [29]. We have expanded our analysis to that of the related family member, OC2, which appears to have a similar function in the development of horizontal cells in the retina. Surprisingly, even though OC2 was expressed in fewer horizontal cell progenitors (Wu et al., 2012), it led to a very similar reduction in the number of horizontal cells in adult KO mice. Studies of mice where both OC1 and OC2 are conditionally removed from the retina will allow for a further dissection of the relative contribution of each family member.

Although loss of either OC1 or OC2 affects the developing retina in similar ways, leading to a drastic loss of horizontal cells, our study also shows that the absence of either of these factors leads to additional phenotypes in the retina. Our microarray analyses allow for an unbiased examination of numerous transcriptomic changes upon loss of these two family members. For instance, transcriptome analysis of E14.5 OC1-KO mice revealed that the expression of Opn4, the photopigment seen in intrinsically-photosensitive ganglion cells, is increased upon OC1 loss. It is curious that the upregulation of Opn4 is transient and the identification of other factors that may compensate for the loss of OC1 warrants future study. The increase in Opn4, along with the observed upregulation of gephyrin and Sox4 and downregulation of Irx4 and Scratch2, suggests that OC1 may play a previously unrecognized role in a subset of ganglion cells (or other retinal cells) during retinal development. Additionally, the upregulation of gephyrin could point to an increase in amacrine cells, as was predicted by the fact that in the absence of OC1, horizontal cell precursors would be expressing the transcription factor Ptf1a (Wu et al., 2013).

Our findings in the OC2-KO mouse contrast with previous findings in the OC1-conditional KO mouse that indicate retinal degeneration begins as early as 5 months of age in OC1-deficient retinas and continues to progress. Although our OC2-KO mice do show an upregulation of genes associated with retinal stress during early adulthood (Figure 6B, C) and a downregulation of some bipolar expressed genes, we do not observe distinctive signs of retinal degeneration until well past one year of age (Figure S2). This lack of degeneration may relate to the viability of our full knockouts in comparison to the conditional knockout mice previously described [29]. As the Onecut transcription factors play integral roles in liver, pancreatic, and immune function [25], [27], [28], a substantial number of our OC2-KO mice and almost all of our OC1-KO mice die soon after birth. Therefore, it may be that those mice that survive to advanced age have activated some compensatory mechanisms or contain some genetic modifiers that allow for a more normal development upon Onecut loss.

Previously we had found that the cone marker thyroid hormone receptor beta 2 (Thrb) was downregulated in the OC1-KO mice [47]. However, Thrb appeared at normal levels by qPCR in the embryonic retinas of the OC2-deficent mice (data not shown). Moreover, we found that other markers of cone photoreceptors, including Gnat2 and Pde6c, appeared normal in the OC2-KO mouse retina, further indicating that cone photoreceptors are not affected in these mice. Surprisingly, we also found that Gnat2 and Prdm1 appeared normal in the embryonic retina of the OC1-KO mouse, despite the downregulation of Thrb. These data may point to a compensatory mechanism operating during retinal development to replace Onecut1 in photoreceptor precursor cells. It may be that other Onecut family members are responsible for this mechanism, but lethality prevents the analysis of compound mutant mice in our background. Further work utilizing additional conditional mouse knockouts will be required to more accurately define the overlapping and distinct roles that the Onecut transcription factors play in retinal development.

## Supporting Information

Figure S1 Expression of adult retinal markers in OC2-KO retinas. In situ hybridization was employed to determine the effects of OC2-deficiency on adult retinal cells. Photoreceptors (A,A’,B,B’,C,C’), bipolar cells (D,D’), amacrine interneurons (E,E’,F,F’,G,G’.H,H’), ganglion cells (I,I’) and Muller glia (J,J’) appear unaffected by loss of OC2. However, the population of horizontal cells is greatly decreased in the absence of OC2 (K,K’). OC2 mRNA deficiency is seen in (L,L’). Scale bars represent 50 µm.
(TIFF)

Figure S2 Retinal degeneration in OC2-KO retinas from aged mice. Immunohistochemistry was performed on WT and OC2 KO retinas from older mice to assess the integrity of the retinas. WT (A,C) and OC2-KO (B,D) littermates were stained with an anti-Rhodopsin antibody (Rho4d2) in red at 12 months of age (A,B) and 16 months of age (C,D). Anti-Calbindin 28k (Calb28k) staining is shown in green to illustrate the loss of horizontal cells in these OC2-KO retinas. Scale bars represent 100 µm.
(TIFF)

Figure S3 Expression of retinal markers in developing E16.5 OC2-KO retinas. *In situ* hybridization was utilized to examine retinal progenitor cells (A,A’), developing photoreceptors (B,B’,C,C’,D,D’), amacrine cells (E,E’), and ganglion cells (F,F’) in WT and OC2-deficient mouse retinas at E16.5. Scale bars represent 200 µm.
(TIFF)

Figure S4 Expression of retinal markers in developing E16.5 OC1-KO retinas. *In situ* hybridization was utilized to examine developing photoreceptors (A,A’,B,B’,C,C’), amacrine cells (D,D’), and ganglion cells (E,E’) in WT and OC1-deficient mouse retinas at E16.5. Scale bars represent 200 µm.
(TIFF)

Tables S1 The gene expression values resulting from Affymetrix microarrays of isolated WT or KO retinas were analyzed using R and the Bioconductor suite. Cel files were background-corrected and normalized using Mas5 and log(2) transformed. Shown are all genes found to be significantly differentially-expressed, where the average values of either WT or KO expression must exceed 7 (to indicate overall expression in either subset of transcriptomic data).
(XLSX)

Table S2 The gene expression values resulting from Affymetrix microarrays of isolated WT or KO retinas were analyzed using R and the Bioconductor suite. Cel files were background-corrected and normalized using Mas5 and log(2) transformed. Shown are all genes found to be significantly differentially-expressed, where the average values of either WT or KO expression must exceed 7 (to indicate overall expression in either subset of transcriptomic data).
(XLSX)

Table S3 The gene expression values resulting from Affymetrix microarrays of isolated WT or KO retinas were analyzed using R and the Bioconductor suite. Cel files were background-corrected and normalized using Mas5 and log(2) transformed. Shown are all genes found to be significantly differentially-expressed, where the average values of either WT or KO expression must exceed 7 (to indicate overall expression in either subset of transcriptomic data).
(XLSX)

Table S4 The gene expression values resulting from Affymetrix microarrays of isolated WT or KO retinas were analyzed using R and the Bioconductor suite. Cel files were background-corrected and normalized using Mas5 and log(2) transformed. Shown are all genes found to be significantly differentially-expressed, where the average values of either WT or KO expression must exceed 7 (to indicate overall expression in either subset of transcriptomic data).
(XLSX)
