# Correction: Onecut1 and Onecut2 Play Critical Roles in the Development of the Mouse Retina

**DOI:** 10.1371/journal.pone.0115786

**Published:** 2014-12-11

**Authors:** The PLOS ONE Staff

The headers for columns M and N in Table S4 are switched. The title for column M should be “avg KO.” The title for column N should be “avg WT.” The correct Table S4 can be viewed below.

## Supporting Information

Table S4 The gene expression values resulting from Affymetrix microarrays of isolated WT or KO retinas were analyzed using R and the Bioconductor suite. Cel files were background-corrected and normalized using Mas5 and log(2) transformed. Shown are all genes found to be significantly differentially-expressed, where the average values of either WT or KO expression must exceed 7 (to indicate overall expression in either subset of transcriptomic data).
(XLSX)
