# Supplementary figures and images for: Onecut1 and Onecut2 Play Critical Roles in the Development of the Mouse Retina
Source: PLoS One. 2014 Oct 14;9(10):e110194. doi: 10.1371/journal.pone.0110194 (PMC4196951; doi:10.1371/journal.pone.0110194)

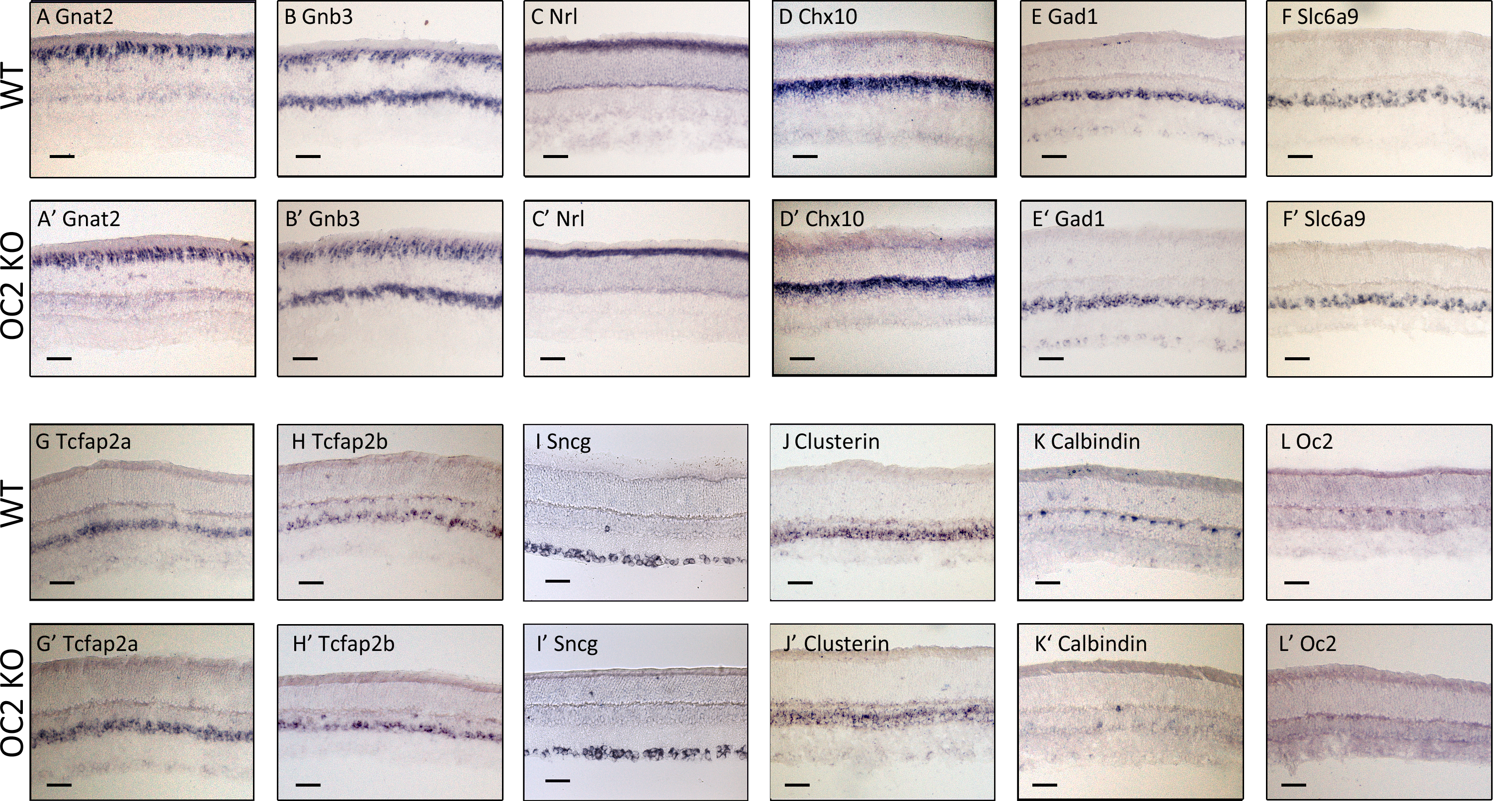

Supplement: Figure S1 — Expression of adult retinal markers in OC2-KO retinas. In situ hybridization was employed to determine the effects of OC2-deficiency on adult retinal cells. Photoreceptors (A,A’,B,B’,C,C’), bipolar cells (D,D’), amacrine interneurons (E,E’,F,F’,G,G’.H,H’), ganglion cells (I,I’) and Muller glia (J,J’) appear unaffected by loss of OC2. However, the population of horizontal cells is greatly decreased in the absence of OC2 (K,K’). OC2 mRNA deficiency is seen in (L,L’). Scale bars represent 50 µm. (TIFF) [file pone.0110194.s001.tiff]

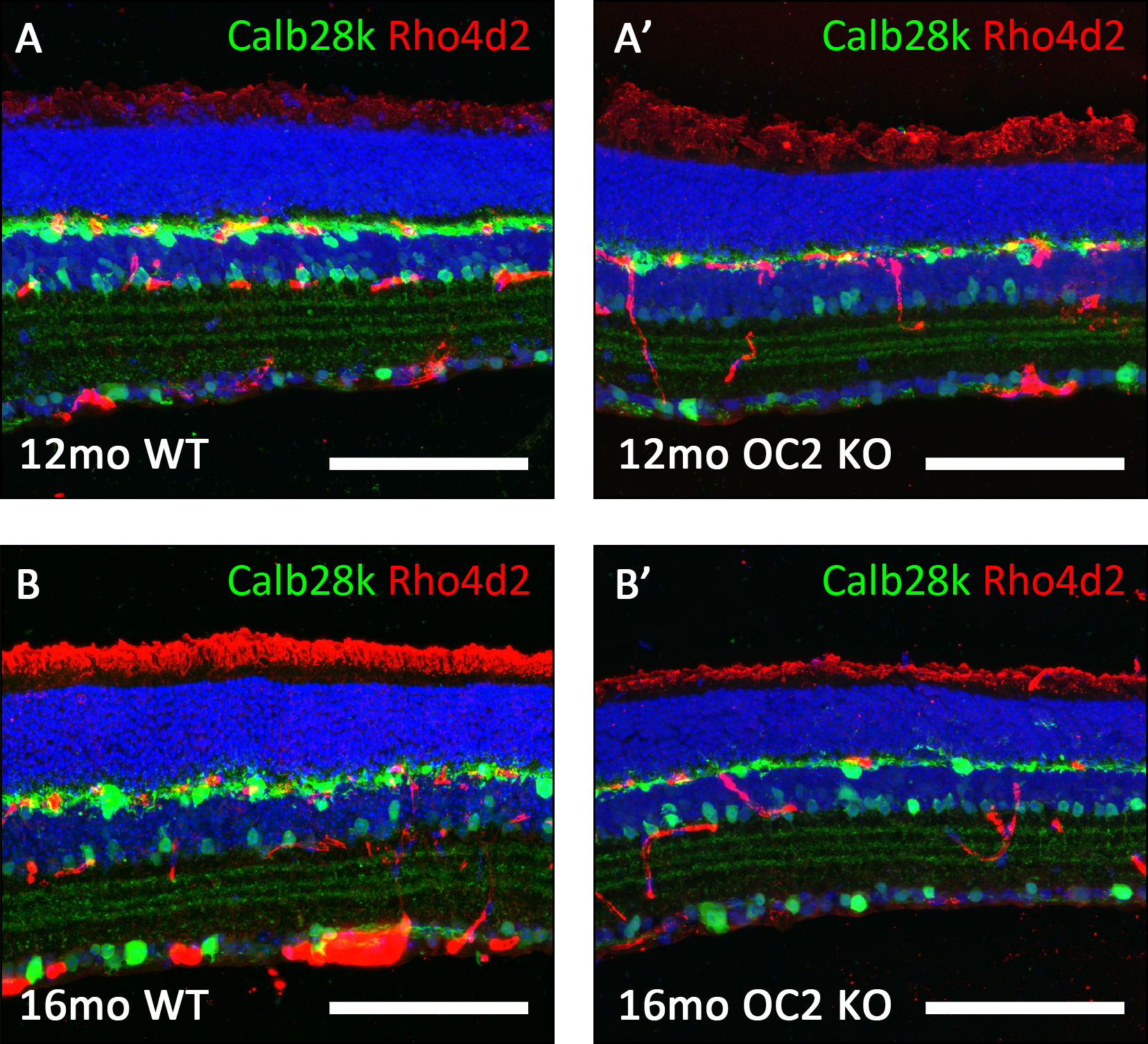

Supplement: Figure S2 — Retinal degeneration in OC2-KO retinas from aged mice. Immunohistochemistry was performed on WT and OC2 KO retinas from older mice to assess the integrity of the retinas. WT (A,C) and OC2-KO (B,D) littermates were stained with an anti-Rhodopsin antibody (Rho4d2) in red at 12 months of age (A,B) and 16 months of age (C,D). Anti-Calbindin 28k (Calb28k) staining is shown in green to illustrate the loss of horizontal cells in these OC2-KO retinas. Scale bars represent 100 µm. (TIFF) [file pone.0110194.s002.tiff]

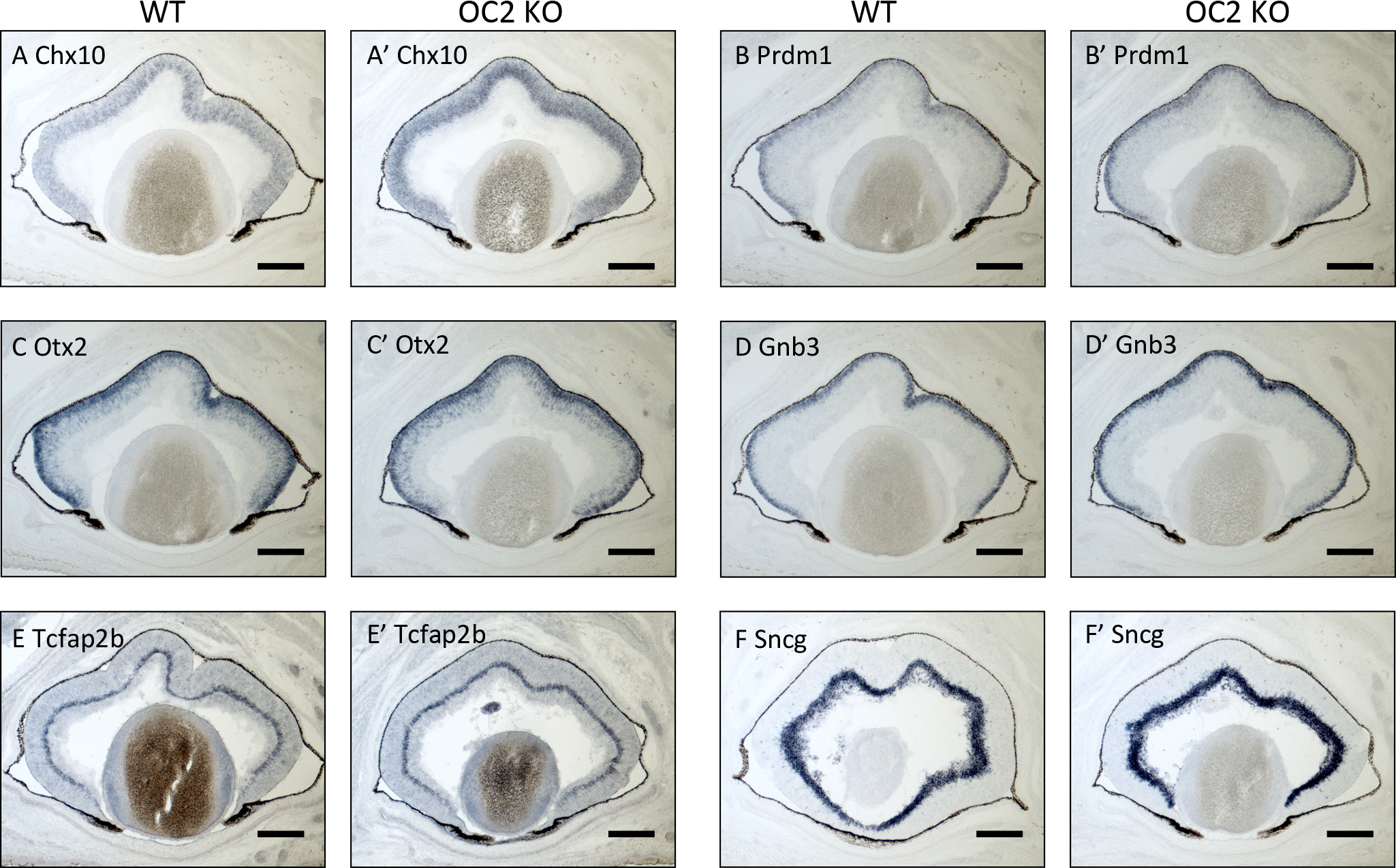

Supplement: Figure S3 — Expression of retinal markers in developing E16.5 OC2-KO retinas. In situ hybridization was utilized to examine retinal progenitor cells (A,A’), developing photoreceptors (B,B’,C,C’,D,D’), amacrine cells (E,E’), and ganglion cells (F,F’) in WT and OC2-deficient mouse retinas at E16.5. Scale bars represent 200 µm. (TIFF) [file pone.0110194.s003.tiff]

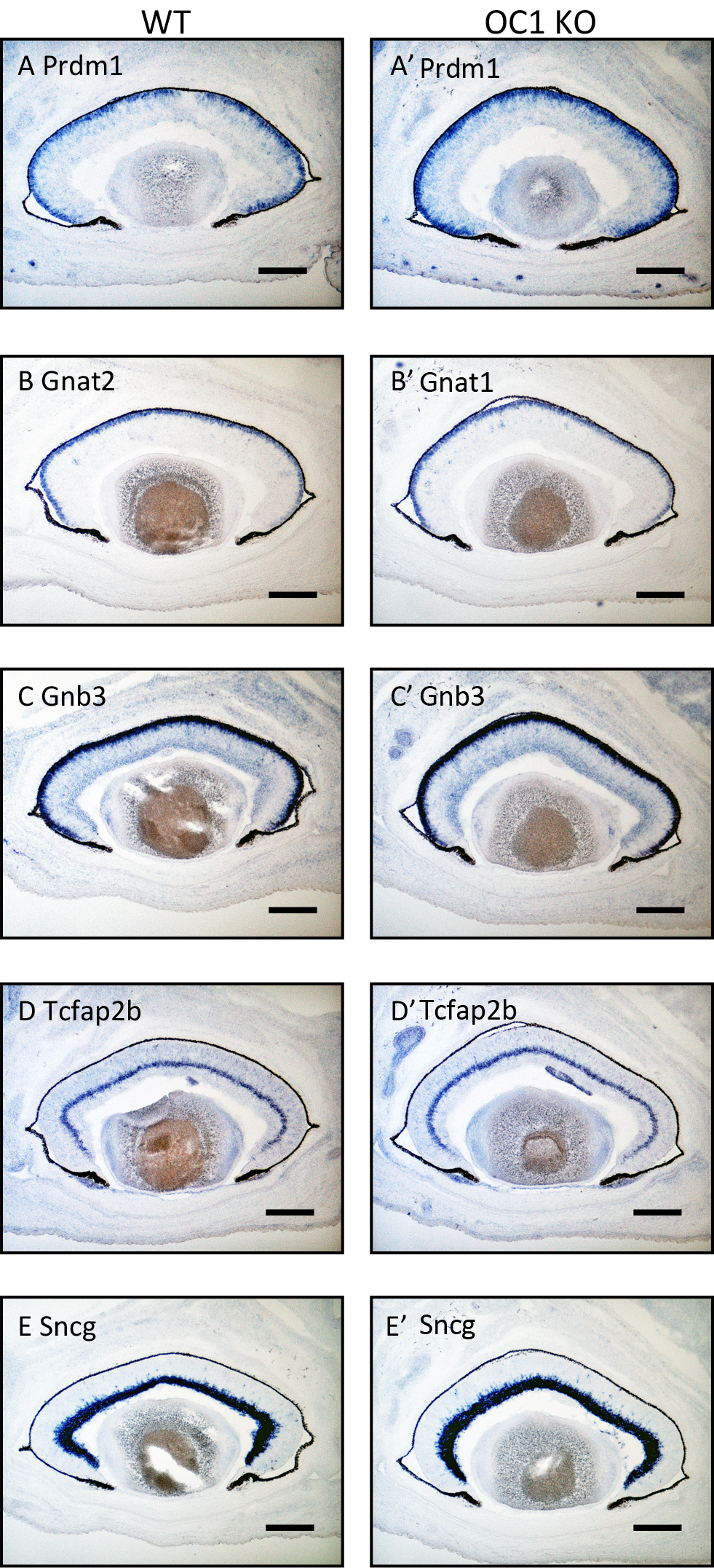

Supplement: Figure S4 — Expression of retinal markers in developing E16.5 OC1-KO retinas. In situ hybridization was utilized to examine developing photoreceptors (A,A’,B,B’,C,C’), amacrine cells (D,D’), and ganglion cells (E,E’) in WT and OC1-deficient mouse retinas at E16.5. Scale bars represent 200 µm. (TIFF) [file pone.0110194.s004.tiff]
